# Dynamic changes in the plasmidome and resistome in the gastrointestinal tract of chickens

**DOI:** 10.1128/spectrum.04074-25

**Published:** 2026-03-26

**Authors:** Marketa Rysava, Katarina Stredanska, Jana Schwarzerova, Marketa Jakubickova, Darina Cejkova, Derya Aytan-Aktug, Saria Otani, Monika Dolejska, Jana Palkovicova

**Affiliations:** 1 Department of Biology and Wildlife Diseases, Faculty of Veterinary Hygiene and Ecology, University of Veterinary Sciences Brno 48358, Brno, Czech Republic; 2 Central European Institute of Technology, University of Veterinary Sciences Brno 48358, Brno, Czech Republic; 3 Department of Microbiology, Faculty of Medicine in Pilsen, Charles University 37740, , Pilsen, Czech Republic; 4 Department of Biomedical Engineering, Faculty of Electrical Engineering and Communication, Brno University of Technology 48274, Brno, Czech Republic; 5 Molecular Systems Biology (MOSYS), Department of Functional and Evolutionary Ecology, Faculty of Life Sciences, University of Vienna 27258, Vienna, Austria; 6 Department of Molecular and Clinical Pathology and Medical Genetics, University Hospital Ostrava 48228, Ostrava, Czech Republic; 7 Research Group for Genomic Epidemiology, National Food Institute, Technical University of Denmark 5205, Kgs. Lyngby, Denmark; 8 Department of Chemistry and Biochemistry, Faculty of AgriSciences, Mendel University in Brno 48269, Brno, Czech Republic; 9 Division of Clinical Microbiology and Immunology, Department of Laboratory Medicine, University Hospital Brno, Brno, Czech Republic; Universita degli Studi dell'Insubria, Varese, Italy

**Keywords:** plasmidome, metagenome, antimicrobial resistance, mobile genetic elements, broiler

## Abstract

The expansion of intensive poultry farming has led to a substantial increase in antibiotic use, which, in turn, has promoted the accumulation of antibiotic resistance genes (ARGs). The chicken gut serves as a reservoir for these genes and provides favorable conditions for their horizontal transfer via mobile genetic elements, such as plasmids. Through this process, commensal bacteria can transfer ARGs to pathogens, facilitating their spread and increasing the risk of transmission to humans. In this study, long-read sequencing was used to characterize the plasmidome and resistome in 12 fecal samples from 3 houses of a commercial broiler chicken farm. All chickens received enrofloxacin in the first days of life, with one house additionally treated with sulfamethoxazole/trimethoprim combination. For comparison, metagenomic analysis using short-read sequencing was performed on the same samples. This study revealed the presence of various ARGs associated with resistance to 25 antibiotic classes. A strong genetic association between MOBP-type plasmids and fluoroquinolone resistance was observed within broiler chicken farms. Temporal trends indicated progressive mobilization of these ARGs, suggesting an increasing potential for horizontal gene transfer. While fluoroquinolone resistance expanded over time, diaminopyrimidine resistance remained stable despite the antibiotic treatment. Most ARGs were carried on small plasmids, and complete plasmid reconstructions ranged from 2.6 to 47.6 kb. Our findings demonstrate that plasmidome sequencing enables high-resolution detection of resistance-associated plasmids that may be overlooked by conventional metagenomic approaches. The observed patterns are consistent with an association between fluoroquinolone use in poultry farms and the presence of plasmid-mediated resistance genes with potential for horizontal dissemination.

**IMPORTANCE:**

Despite the crucial role of plasmids in antimicrobial resistance (AMR) dissemination, studies focusing on plasmidomes, defined as the complete set of plasmids, remain limited. This study is the evidence that chicken farms, where fluoroquinolone treatment is a standard practice, act as an important reservoir of plasmid-mediated antibiotic resistance which may not be revealed by commonly used approaches. Combining a metagenomic approach with a focus on plasmids enhances our ability to understand the genetic context and mechanisms underlying AMR transmission. The findings emphasize the importance of targeted plasmid analysis to improve surveillance and risk assessment of AMR transmission in microbial ecosystems.

## INTRODUCTION

Intensive poultry farming has led to increased antibiotic use, driving the rise of antimicrobial resistance (AMR), which is a major global health concern. Despite the prohibition of antibiotics as growth promoters in EU livestock farming since 2006 and a ban on their use for disease prevention in 2022, more than half of global antibiotic consumption is still attributed to agriculture (1). In the Czech Republic, penicillins, tetracyclines, and sulfonamides are the most commonly administered to livestock (2). While fluoroquinolone antibiotics are not among the top antibiotic classes by total weight, they are the most frequently administered antimicrobial group in the poultry sector which accounts for almost 80% of fluoroquinolone consumption in livestock. Although sales of fluoroquinolones fluctuated between 2011 and 2022, there was a slight overall increase from 1.5 mg/Population Correction Unit (PCU) in 2011 to 1.6 mg/PCU in 2022. During the implementation of the second Czech national action plan on antimicrobial resistance (2019–2022), fluoroquinolone consumption decreased by 13% (2, 3). In contrast, fluoroquinolones are not approved in poultry farming in the United States (4) and Australia (5).

Despite the beneficial roles in immune development, pathogen exclusion, and host nutrition, the gastrointestinal microbiome can also serve as a reservoir of antibiotic resistance genes (ARGs). These genes may be transferred between bacteria via horizontal gene transfer (HGT), further facilitating the persistence and spread of AMR within the host and its surroundings (6). Approximately 60% of human infectious diseases are estimated to be caused by zoonotic pathogens capable of carrying ARGs (7). The resistant bacteria originating from the poultry gut may be transmitted to humans through the food chain, particularly via contaminated chicken meat (8) or introduced into the environment through the application of chicken manure as fertilizer (7).

Plasmids play a central role in HGT (9). They are characterized by significant variability, their size ranging from a few kilobases to hundreds of kilobases, and their copy number differing markedly between cells. Despite this characteristic, plasmid DNA (pDNA) constitutes only a small fraction of the total cellular DNA compared to chromosomal DNA (10, 11). The plasmidome, defined as the total collection of plasmids in a specific environment, plays a significant role in microbial evolution and adaptation (12).

The number of studies focusing on the plasmidome, particularly in the context of the chicken gut, is limited. Previous publications were focused on the plasmidome in sewage (12–15) or in the gut of other animals (16, 17); however, the role of plasmidome in the spread of AMR in chicken gut remains largely unexplored. The focus of this research was to characterize plasmidome and resistome in the fecal droppings of chickens, as well as to obtain complete plasmid sequences and link them to specific resistance genes using long-read sequencing technology. This was followed by tracing the temporal changes in the plasmidome to investigate the dynamics and evolution of plasmid-mediated AMR during the broiler chicken’s lifetime in respect to the use of antibiotics.

## MATERIALS AND METHODS

### Case presentation

Twelve samples of fecal droppings were obtained from Ross 308 chicken (a commercial broiler breed) flocks located in three houses, with up to 21,000 chickens per house. From each house, sampling began in the second week of life and continued weekly for 3 weeks until the birds reached slaughter age. Cohorts arrived on Saturday, Wednesday, or Thursday depending on farm logistics. However, sampling was performed uniformly on Fridays to ensure consistent temporal alignment across groups. A composite fecal sample from each house was collected each week. The uric acid layer was manually removed from the chicken droppings to prevent potential inhibition of DNA extraction, and each sample was stored at −20°C.

Chickens in all houses were treated with enrofloxacin in the first days of life (10 mg/kg body weight/day) starting on day 3 and administered for four consecutive days. One of the houses (House 2) was subsequently treated with a sulfamethoxazole/trimethoprim combination due to higher mortality. Animals received 7.5 mg/kg trimethoprim plus 37.5 mg/kg sulfamethoxazole per day for three consecutive days, administered in drinking water. No additional antimicrobials, pharmaceuticals, disinfectants, metals, or other chemical agents were administered during the study period, minimizing potential confounding exposures.

### DNA extraction and post-extraction treatment for long-read sequencing

Total pDNA was extracted from the obtained samples using Plasmid Mini Kit (Qiagen, GE) and Plasmid Midi Kit (Qiagen, GE) to obtain both small and large plasmids. The extractions were performed in accordance with the manufacturer’s guidelines with modifications provided in Supplemental material. After extraction, residual chromosomal and environmental DNA were removed using Plasmid-Safe ATP-Dependent DNase (10 U) (Biosearch Technologies, UK), and pDNA was subsequently amplified using NxGen phi29 DNA Polymerase (10 U) (Biosearch Technologies, UK). This could introduce technical bias addressed in Supplemental material; however, all samples were processed identically. A detailed protocol for post-extraction treatment is provided in Supplemental material. Treated pDNA from the Plasmid Mini Kit and Plasmid Midi Kit was mixed 1:1 for each sample.

### DNA extraction for short-read sequencing

A total of 250 mg of droppings was used for DNA purification using the QIAamp PowerFecal Pro DNA Kit (QIAGEN, GE), following the manufacturer’s protocol with minor modifications. Sample homogenization was carried out using the bead-beating step provided in the kit, employing the Vortex-Genie 2 and horizontal adapter (Scientific Industries, USA). DNA was eluted following a 5 min incubation with the provided elution buffer. DNA yield was quantified using a NanoPhotometer N60 (Implen, GE), and DNA integrity was assessed by running a 1% agarose gel.

### Plasmidome and metagenome sequencing

For long-read sequencing of plasmidome, DNA libraries of each sample were prepared from the treated pDNA using the SQK-LSK114 ligation kit (Oxford Nanopore Technologies, ONT, UK). The manufacturer’s guidelines were followed, with modifications specified in Supplemental material. Each DNA library was loaded onto a FLO-PRO114M flow cell, one sample per flow cell, and sequenced on the P2Solo long-read sequencing platform (ONT, UK).

For short-read sequencing, DNA was diluted to a concentration of 10 ng/µL. DNA libraries were prepared from 10 ng of metagenomic DNA using VAHTS Universal Plus DNA Library Prep Kit for Illumina (Vazyme Biotech, China) and sequenced via paired-end chemistry (PE150) on the NovaSeq X platform (Illumina, USA) by Biomarker Technologies Co., Ltd. (Beijing, China). The average yield per sample was 12.03 Gb of sequencing data, with the percentage of Q30 bases in each sample exceeding 91.89%.

### Long-read data analysis

Long-read sequencing data were adapter- and quality-trimmed (Supplemental material), while no further filtering to remove chromosomal DNA was performed, as some regions can occur in both, plasmids and chromosomal sequences, making the separation unreliable. To address technical limitations (Supplemental material), GraphPad Prism v10.0.0 (GraphPad Software, Boston, USA) was used for subsequent data comparison (Supplemental material), and the statistical analysis was performed using Pearson correlation, with a significance threshold set at *P* ≤ 0.05.

KMA tool v1.4.15 (18) with option “bcNano” was used to compare the genetic content of processed reads. The ARGs were assessed using PanRes v1.0.1 database (19). The mobile genetic elements (MGEs), besides plasmids, were identified by the MGE v1.0.2 database (20), and the plasmid content was analyzed by mapping to the *oriT* database of the MOB-suite v3.1.9 (21). To obtain comparable results across different samples, the relative abundance was calculated and normalized using Reads Per Kilobase Million (RPKM) value. Twenty most abundant MGEs and plasmids were categorized as distinct groups, while the remaining elements were consolidated into an “others” group. The resulting data were visualized using the Matplotlib library v3.4.4 in Python (https://github.com/janpal-cz/Plasmidome).

To further investigate the co-occurrence of MGEs and ARGs across houses and time points, a network-based approach was employed (22). Seven datasets (Table 1) were generated from the alignment files produced by the KMA tool for ARGs and plasmids.

**TABLE 1:** Data sets used for network analysis

Network	Data set
House 1	H1_13d, H1_20d, H1_27d, H1_35d
House 2	H2_10d, H2_17d, H2_24d, H2_31d
House 3	H3_9d, H3_16d, H3_23d, H3_30d
Time point 1	H1_13d, H2_10d, H3_9d
Time point 2	H1_20d, H2_17d, H3_16d
Time point 3	H1_27d, H2_24d, H3_23d
Time point 4	H1_35d, H2_31d, H3_30d

Count-transfer-matrix, obtained as described (Supplemental material), was used for the construction of an undirected network which was visualized in Cytoscape v3.9.0 (23). As the network represents node co-occurrence and not their similarity, node positions were manually adjusted for improved clarity. However, the network retains the original connectivity.

Plasmids carrying ARGs were assembled and corrected (Supplemental material). The assemblies were analyzed using the reconstruction mode of MOB-suite (21). The ARGs were identified by comparing the assemblies to the PanRes v1.0.1 database using ABRicate (https://github.com/tseemann/abricate) with the same criteria as in the raw read analysis. Plasmids carrying ARGs were annotated using Bakta v1.10.3 (24) with subsequent manual inspection and correction in Geneious Prime 2024.0.7 (http://www.geneious.com/). The plasmids were also compared with PLSDB v. 2024_05_31_v2 (25) (accessed on November 2024) to assess their relatedness and dissemination potential compared to known plasmids.

### Short-read data analysis

Short metagenomic sequencing reads were pre-processed (Supplemental material). Trimmed, contamination-free sequencing reads were used for taxonomic profiling with MetaPhlAn v4.0.6 (26) and the embedded GTDB release 207 (27). Taxonomic profiles were visualized as a heatmap with Python Matplotlib library v3.4.4 using a script published within GitHub repository for this manuscript (https://github.com/janpal-cz/Plasmidome). Genetic content (ARGs, MGEs, and plasmids) of the processed reads was assessed using the KMA tool v1.4.15 with option “1t1” and databases PanRes v1.0.1, MGE v1.0.2, and MOB-suite v3.1.9 with subsequent analysis as described for long-read processing of genetic content.

## RESULTS

### Plasmids associated with antibiotic resistance genes

The assembly of all 12 plasmidome samples produced a high number of contigs and showed a high proportion of non-circular plasmid fragments as well as pieces of chromosomes (Table S1). The number of contigs was ranging from 169 to 9,818 per sample. Frequently, the plasmid fragments appeared to represent duplicated plasmids within a single contig. This was supported not only by several repetitions of a single plasmid marker (e.g., plasmid replicon) but also by post-assembly mapping of corresponding reads. Based on the assembly analysis, it was revealed that 121 out of 215 identified ARGs (56.3%) were localized on chromosomal fragments (Table S1).

**Fig 1:**
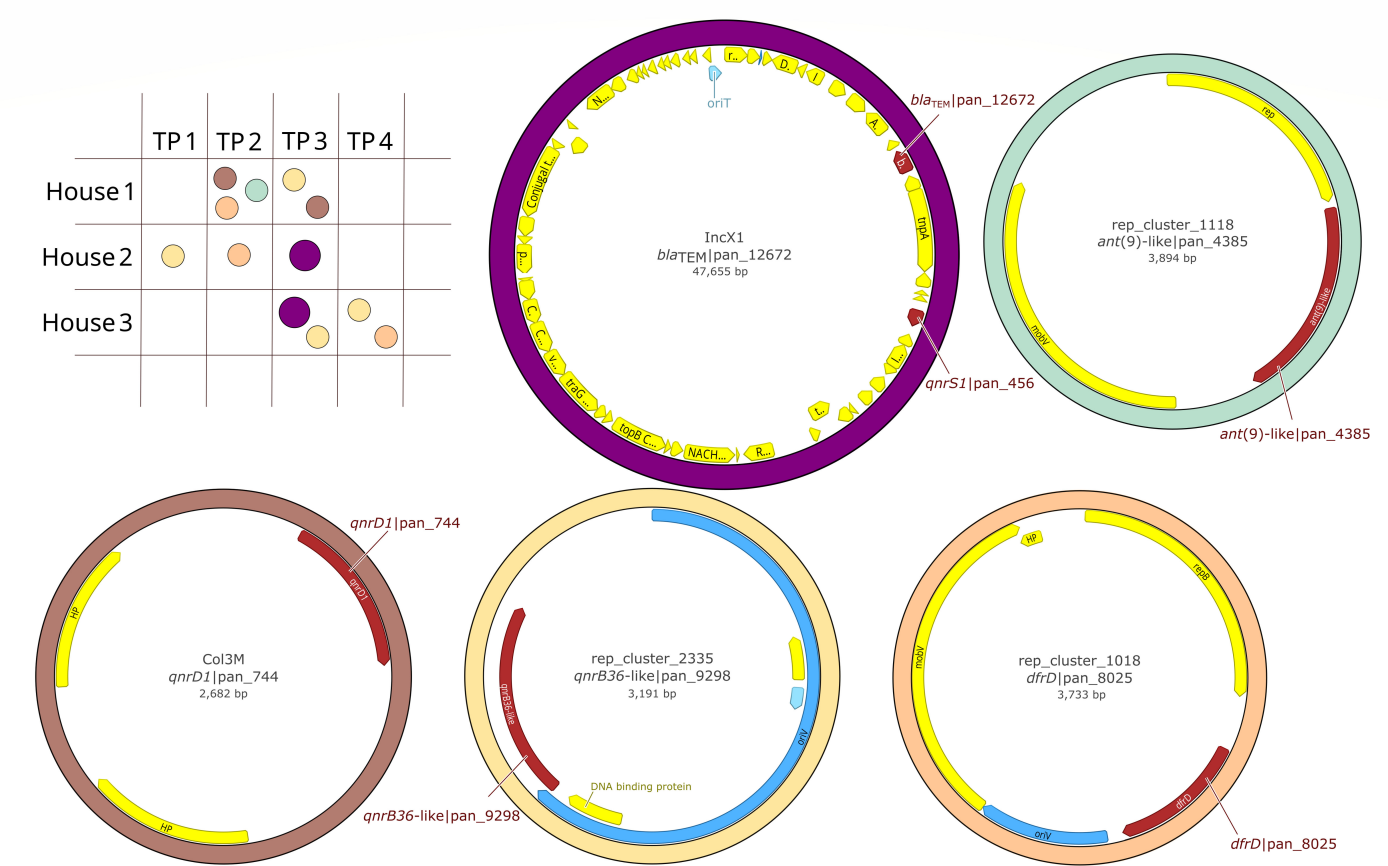
Complete plasmids associated with antibiotic resistance in samples from a chicken farm. The diagram (top left) indicates the presence of selected plasmids in individual samples collected from the three different houses (Houses 1–3) across four time points (TP1–TP4). Circular maps illustrate the annotated genetic structure of five representative plasmids assembled from long-read sequencing data, with antibiotic resistance genes (highlighted in dark red), origin of transfer (blue), and additional coding sequences (yellow).

Across all assembled samples, ARGs to various groups of antibiotics were detected and differentially distributed between chromosomal and plasmid fragments, with fluoroquinolone ARGs primarily associated with plasmids. ARGs to 18 different groups of antibiotics were detected (Table S1). The multidrug resistance (MDR)-conferring ARGs were strictly associated with chromosomal sequences, while ARGs to lincosamides were present in both chromosomal and plasmid contigs. On the other hand, ARG variants for fluoroquinolones were mostly (93.75%, 45/48) linked to plasmid fragments. We reconstructed five complete plasmids carrying ARGs ranging from 2.6 to 47.6 kb in size (Fig. 1). Many ARGs were localized on linear plasmid fragments which could not be fully reconstructed. Many candidate plasmids contained no ARGs. In 2 (H1_35d, H2_31d) out of 12 samples, no ARGs were identified in assemblies, while in 3 other samples (H1_13d, H3_9d, and H3_16d), all present ARGs were on chromosomal fragments (Table S1).

#### Plasmid rep_cluster_2335 carrying qnrB36-like|pan_9298

The most occurring plasmid across the samples was a 3 kb long MOBP-like plasmid of rep_cluster_2335 containing *qnrB36*-like|pan_9298. This plasmid was found at different time points among all three houses from the same predicted host (*Escherichia coli*). The plasmids slightly varied in size, with 3,001 bp in H2_10d (PX387659), 3,071 bp in H3_23d (PX387656), and 3,191 bp in H1_27d and H3_30d (PX387658 and PX387657). The occurrence of the plasmid spiked in the H3_30d with coverage 88,543 which was also responsible for a huge increase in fluoroquinolone resistance observed in raw read plasmidome analysis. The comparison of 3,191 bp long plasmid from H3_30d with PLSDB showed a high similarity (99.96% similarity) with a previously published plasmid (OQ787038.1) from *E. coli* isolated from Eurasian coot and the 3,071 bp plasmid from H3_23d with plasmid (CP043749.1, similarity 99.96%) from *Salmonella enterica* isolated from .

#### Plasmid Col3M carrying qnrD1|pan_744

Another ARG for fluoroquinolone resistance was *qnrD1*|pan_744 carried by a small 2.6 kb long Col3M plasmid present in H1_20d and H1_27d (PX399215 and PX399216). These plasmids were identical, and the predicted host was *Proteus mirabilis*. The previously described plasmids (AP024494.1 and CP057825.1) of highest similarity (99.96% and 99.90%, respectively) were associated with *Providencia vermicola* from human sample in Nepal and *Citrobacter freundii* from pig feces in the United Kingdom.

#### Plasmid IncX1 carrying qnrS1|pan_456 and bla_TEM_|pan_12672

The *qnrS1*|pan_456 was co-harbored with *bla*_TEM_|pan_12672 on a 47.6 kb long IncX1 plasmid (Fig. 1). This plasmid was found at the third collection point in house 2 (PX387660) and house 3 (PX387661). The plasmids were highly similar, with pairwise identity 99.8%. Both plasmids were highly similar (coverage 98.2%, similarity 99.91%) to a previously described plasmid (MH121702.1) from Czech Republic found in rook feces. However, these plasmids were also identified in human bloodstream infections, pig feces, and broiler samples (CP115825.1, CP122865.1, and LR882060.1) from diverse countries isolated in different years. All of these plasmids are associated with *E. coli* strains.

#### Plasmid rep_cluster_1018 carrying dfrD|pan_8025

A 3.7 kb long plasmid rep_cluster_1018 containing *dfrD*|pan_8025 was identified in H1_20d (PX387668), H2_17d (PX387669), and H3_30d (PX387670). The most similar plasmid (NZ_CP120612.1) was from *Bacillus subtilis* which is 8.7 kb long and contains *tet*(M) and *tet*(L) genes instead of *dfrD*. The plasmids were 100% identical with coverage 80% which corresponds to the difference in the ARGs content.

#### Plasmid rep_cluster_1118 carrying ant(9)-like|pan_4385

The last fully assembled plasmid was 3.9 kb long, with replicon rep_cluster_1118 of MOBV group originating from *Staphylococcus aureus* which carried *ant (9*)-like|pan_4385, also known as a gene *spd*. This plasmid was fully assembled only in H1_20d (PX399214). Contigs co-harboring rep_cluster_1118 and *ant (9)-*like|pan_4385 were also present in H2_17d and H3_30d. Nevertheless, these contigs were linear, often larger than the fully assembled plasmid, and it was not possible to reliably reconstruct a complete plasmid.

### Plasmid-ARG interactions across the chicken farm

**Fig 2:**
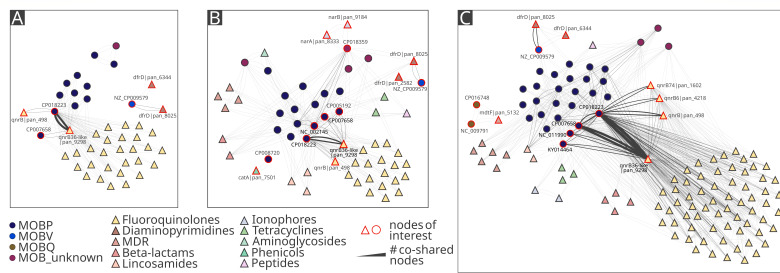
Co-occurrence network of antibiotic resistance genes and plasmids in different houses, with (**A**) corresponding to house 1, (**B**) to house 2 and (**C**) to house 3. The undirected network, where nodes represent plasmids (circles) or antibiotic resistance genes (triangles) and edges represent their co-occurrence on the same sequence. The width of the edges corresponds to the number of sequences co-sharing the specific nodes ranging from one read to 20,712 reads. The layout does not represent similarity or spatial distance as node positions were manually adjusted for an improved visual clarity.

The associations found between ARGs and plasmids pointed out a key role of plasmids in the spread of ARGs to the following classes of antibiotics: fluoroquinolones, diaminopyrimidines, MDR, beta-lactams, lincosamides, ionophores, tetracyclines, aminoglycosides, phenicols, and peptides (Fig. 2).

Using network-based co-occurrence analysis, a strong genetic association between MOBP-like plasmids and fluoroquinolone ARGs was revealed within the chicken farm (Fig. S1). At the early time points, *qnr* genes were present but showed only moderate plasmid connections (Table S2). However, their interactions expanded substantially in the following weeks, involving a broader range of genes and plasmids. Plasmid CP018223|MOBP showed the most prominent co-localization with *qnrB36*-like|pan_9298 (20,712 edges). However, this plasmid was also frequently connected to other fluoroquinolone resistance genes, such as *qnrB*|pan_498 (273 edges), *qnrB74*|pan_1602 (137 edges), and *qnrB6*|pan_4218 (105 edges). All of these co-localizations were detected in the house 3 data set (Table S2).

**Fig 3:**
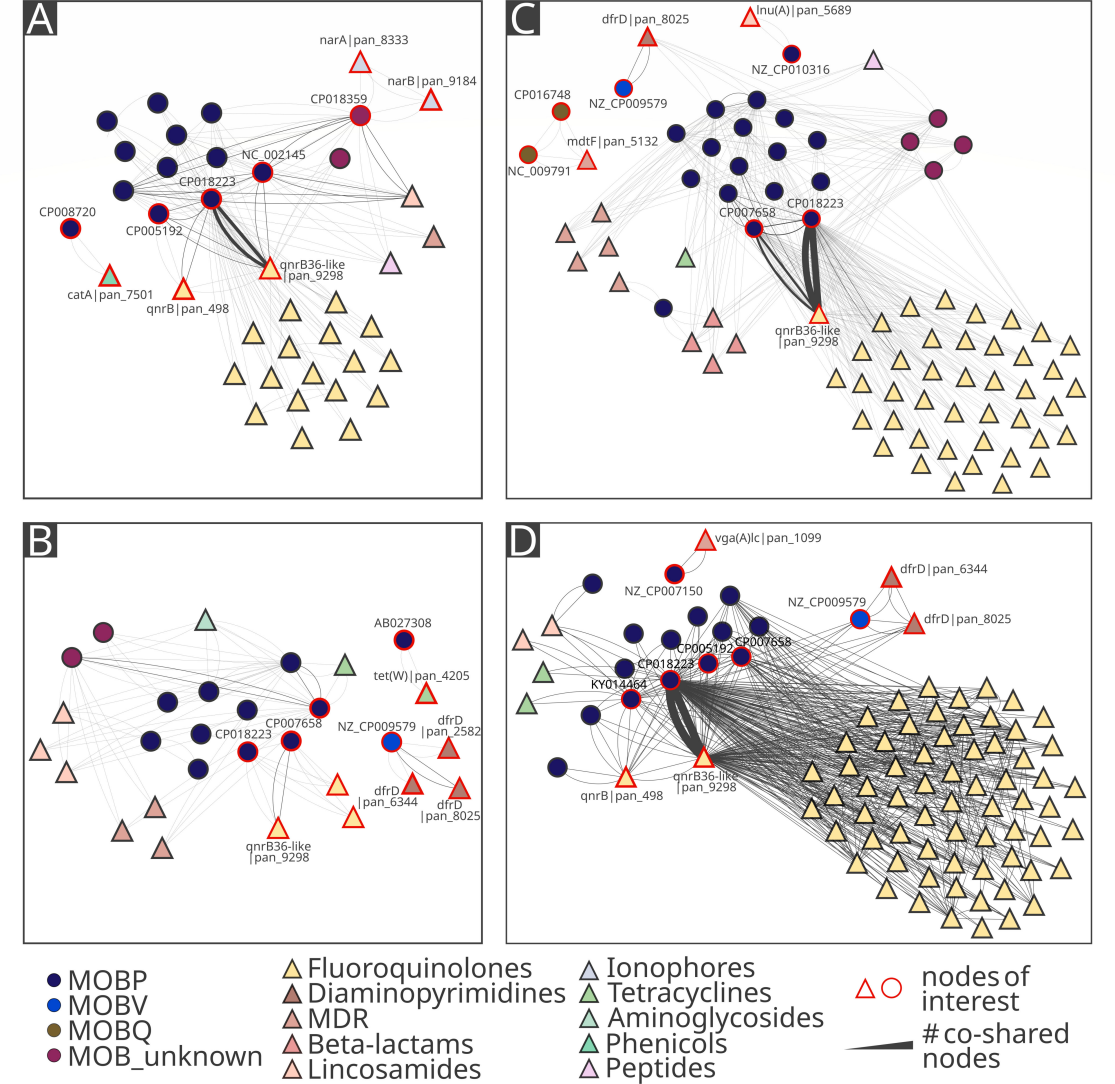
Co-occurrence network of antibiotic resistance genes and plasmids in different time points, with (**A**) corresponding to time point 1, (**B**) to time point 2, (**C**) to time point 3, and (**D**) to time point 4. The undirected network, where nodes represent plasmids (circles) or antibiotic resistance genes (triangles) and edges represent their co-occurrence on the same sequence. The width of the edges corresponds to the number of sequences co-sharing the specific nodes ranging from one read to 20,253 reads. The layout does not represent similarity or spatial distance as node positions were manually adjusted for an improved visual clarity.

Beyond its dominant association with plasmid CP018223|MOBP, the *qnrB36*-like|pan_9298 gene was also detected on plasmid CP007658|MOBP across all time points though at much lower frequencies (3, 13, 234, and 466 edges). Across the time points, *qnrB36*-like|pan_9298 additionally appeared on a variety of other plasmids (1–362 edges) (Fig. 3).

Diaminopyrimidine ARGs showed much more stable and less extensive plasmid associations compared with *qnr* genes. These were consistently dominated by *dfrD|*pan_8025 and plasmid NZ_CP009579|MOBV across all houses, with number of edges increasing from house 1 (11 edges) to house 2 (33 edges) and peaking in house 3 (102 edges). Other *dfr* variants showed only sporadic associations.

In time point 1, no plasmid-*dfr* co-localizations were detected. Three *dfr* variants (*dfrD*|pan_2582, *dfrD*|pan_6344, and *dfrD*|pan_8025) co-occurred exclusively with plasmid NZ_CP009579|MOBV in time point 2, with different number of edges (1, 2, and 37, respectively). At time point 3, the dominant variant *dfrD*|pan_8025 was associated primarily with NZ_CP009579|MOBV (13 edges). However, minor additional interactions (one to two edges per interaction) were observed with three other plasmids (Fig. 3A through C. At time point 4, NZ_CP009579|MOBV remained the main plasmid associated with *dfr* genes (93 edges with *dfrD*|pan_8025 and 2 edges with *dfrD*|pan_6344), while only one other plasmid showed a minimal co-localization signal (2 edges) (Fig. 3D).

Other plasmid-ARG associations were less frequent and house-specific (Fig. 2). In house 2, two genes encoding resistance to ionophores (*narA*|pan_8333, *narB*|pan_9184) co-localized with plasmid CP018359|MOB_unknown and *catA*|pan_7501 was associated with plasmid CP008720|MOBP. In house 3, two MOBQ plasmids (NC_009791|MOBQ and CP016748|MOBQ) were associated with the *mdtF*|pan_5132 gene, which provides MDR (Fig. 2 and 3).

### Mobilome

#### Mobile genetic elements

The analysis of MGEs, excluding plasmids, showed a wide variety of identified elements, including miniature inverted-repeat transposable element (MITE), insertion sequence (IS), mobilizable transposon (MTn), and transposon (Tn) (Table S3).

The diversity of MGE groups in the metagenome was comparable to that observed in the plasmidome. In both data sets, the most abundant insertion sequence was IS*Ljo1*, which was present at all time points and in all houses. However, MITE*Ec1*, IS*Enfa4,* and IS*679* exhibited lower abundance in metagenome (Fig. S2).

Metagenomic MGE abundance was relatively uniform (316.3–657.8 RPKM) across the whole data set, whereas the abundance of MGE within plasmidome was varying across sampling points. The most pronounced difference between the two datasets occurred in H3_30d, where plasmid-associated MGE abundance reached 861.0 RPKM, while the MGE in metagenomic data set comprised 80.8 RPKM (Table S3).

#### Plasmids

**Fig 4:**
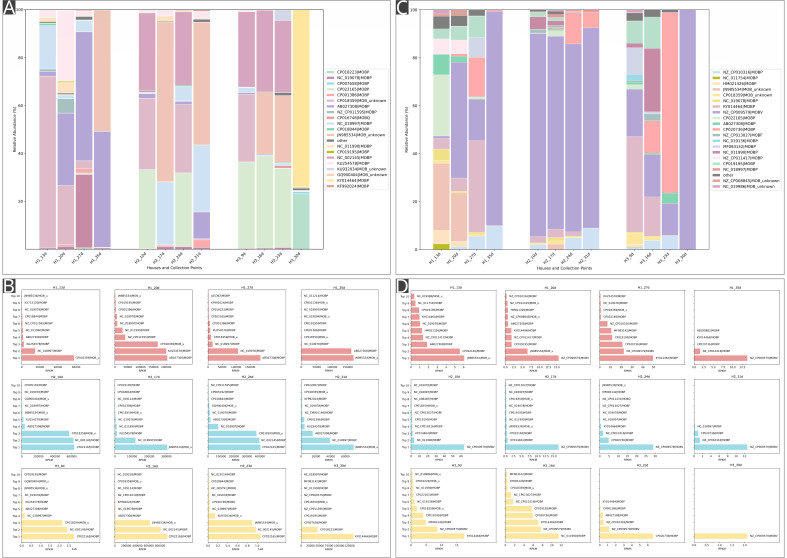
Relative abundance and distribution of plasmid types across different sampling points and houses. Stacked bar plots show the relative abundance of various plasmid types in the plasmidome (**A**) and metagenome (**C**) fractions of three chicken houses (H1, H2, H3), each sampled at four different time points. Because of the plasmid diversity, each bar plot has its own color code specified in the legend. The top 20 plasmid groups were categorized separately, and remaining plasmids were grouped as “others.” Horizontal bar charts present the top 10 most abundant plasmid types identified in each sample from the plasmidome (**B**) and metagenome (**D**).

Plasmids detected in the plasmidome analysis exhibited significantly higher diversity compared to metagenomic analysis (Fig. 4A and C). Across the entire plasmidome data set, 133 different plasmid types were detected, with the number of plasmid types ranging from 20 to 75 per sample. In comparison, the metagenome comprised 59 unique plasmid types, with per-sample diversity ranging from 1 to 31 (Table S3). Plasmid abundance in the plasmidome showed extensive between-sample variability, ranging from 8.0 × 10⁴ to 7.7 × 10⁶. In contrast, metagenomic plasmid abundance remained low (2.10–129.48 RPKM) and showed only limited variation across sampling points.

Notably, a single plasmid (NZ_CP009579|MOBV) was detected in the H3_30d metagenome, and its abundance remained very low (2.10 RPKM) (Fig. 4D), whereas the corresponding plasmidome sample contained a total of 50 unique plasmid types, with the total abundance of 191,401.23 RPKM (Fig. S3). Across all houses, plasmid abundance did not show a consistent temporal increase and instead fluctuated substantially, especially in the plasmidome (Table S3).

The most prevalent plasmids in the plasmidome were CP018359|MOB_unknown, CP022165|MOBP, and NC_002145|MOBP. All three plasmids reached their respective abundance peaks at the H3_9d sampling point (2,160,859.69; 2,777,685.51; 2,417,088.66 RPKM). CP018359|MOB_unknown and CP022165|MOBP were also detected in the metagenomic data set, but at substantially lower abundances (2.02 RPKM and 0.89 RPKM in H3_9d). NC_002145|MOBP was not detected in the metagenome at any sampling point (Table S3).

Temporal shifts in plasmid composition revealed that certain plasmids, such as KY014464|MOBP and JN985534|MOB_unknown, persisted across samples in plasmidome and were also detected in the metagenome (Fig. 4B and D).

### Antibiotic resistance genes

**Fig 5:**
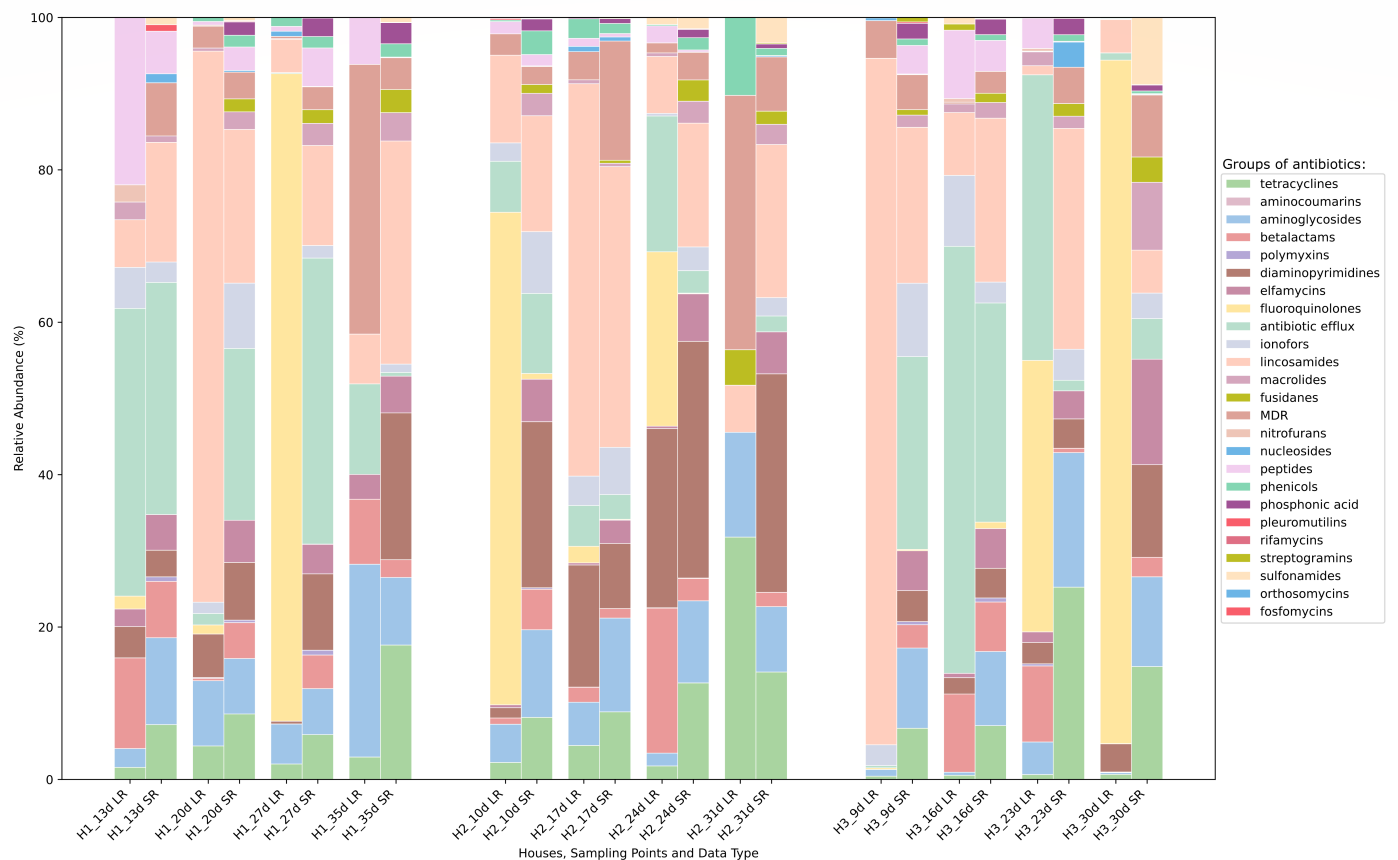
Relative abundance and distribution of ARGs across different sampling points and houses. Stacked bar plot displays differences in relative abundance of genes conferring resistance to 25 groups of antibiotics in the three houses (H1, H2, H3) of a commercial chicken farm in Czech Republic collected within four time points between two datasets: the plasmidome, obtained by long-read sequencing (LR), and the metagenome, obtained by short-read sequencing (SR). MDR—multidrug resistance.

The identified ARGs in plasmidome and metagenome were associated with 25 groups of antibiotics (Fig. 5; Table S3).

Relative abundance and variants of *qnr* genes were highly diverse in the plasmidome, where a total of 79 distinct *qnr* variants were detected, while only two *qnr* variants were identified in the metagenomic data set, both of which were also present in the plasmidome (Table 2; Table S3).

**TABLE 2:** Total abundances of *qnr*, *dfr,* and *sul* genes in plasmidome and metagenome

Sampling point	Abundance of ARGs (plasmidome/metagenome) in RPKM		
	qnr	dfr	sul
H1_13d	1.9/0.0	4.5/7.69	0.0/2.1
H1_20d	2.4/0.0	11.3/14.81	0.0/0.8
H1_27d	293.9/0.1	1.2/40.78	0.0/0.3
H1_35d	0.0/0.0	0.0/25.13	0.0/0.8
H2_10d	256.1/1.9	5.4/54.31	0.2/0.1
H2_17d	6.1/0.2	45.0/14.46	0.0/0.0
H2_24d	31.2/0.1	32.1/36.57	1.3/1.7
H2_31d	0.0/0.0	0.0/28.83	0.0/3.4
H3_9d	0.7/0.4	0.0/9.5	0.0/0.0
H3_16d	0.0/1.7	2.8/7.7	1.1/0.2
H3_23d	162.9/0.0	12.8/2.35	0.4/0.1
H3_30d	5862.9/0.0	241.4/7.25	1.1/5.2

Although distinct *qnr* genes were identified in plasmidome samples from each house following enrofloxacin treatment, the abundance patterns were strongly dominated by the *qnrB36-*like| pan_9298 and occurred within each sampling point from each house in the plasmidome data set. Although the variant predominated within each house, these maxima appeared at different sampling points rather than forming a continuous temporal increase. The overall maximum was observed in H3_30d (5597.9 RPKM). Of the 79 plasmidome *qnr* variants, 33 were detected exclusively in the H3_30d sample, while the corresponding metagenomic sample did not contain any *qnr* genes. In the metagenome, *qnrB36-like*| pan_9298 was detected only once (H3_9d, 0.038 RPKM), and the only other variant present was *qnrS1*|pan_456, which appeared in all houses but at low abundance (0.12–1.87 RPKM) (Table S3).

Diverse *dfr* variants were identified in both metagenome and plasmidome data set (Table S3). In the plasmidome, the treated house 2 displayed higher *dfr* abundances than the other two houses (Table 2), apart from the outlier represented by H3_30d, with 11 distinct *dfr* variants detected in H2, compared to 7 variants in H3 and 5 in H1 (Table S3). Across sampling points within the metagenome data set, the abundance of *dfr* genes varied moderately (Table 2), 14 *dfr* variants were identified in H1, 13 variants in H2, and 10 in H3 (Table S3). The *dfrD*|pan_8025 variant was predominant in both data sets. Even though the abundance was similar across most sampling points, a pronounced peak in plasmidome was observed in sampling points H2_17d (40.8 RPKM), H2_24d (26.5 RPKM), and especially in H3_30d, where this variant was responsible for 95.8% of the *dfr* abundance (231.3 RPKM of a total 241.4 RPKM). In the metagenome, the *dfrD*|pan_8025 variant reached its highest values in H2_10d (43.14 RPKM), H2_24d (30.25 RPKM), and H1_27d (32.38 RPKM). The peak of *dfrD*|pan_8025 observed in the H3_30d of plasmidome data set was not discovered in the metagenome, *dfrD* abundance reached only 25.5% (1.85 RPKM of a total 7.25 RPKM).

Notably, sulfonamide resistance genes remained at low or undetectable levels both in the plasmidome and the metagenome (Table 2).

### Association of observed results with bacterial species

**Fig 6:**
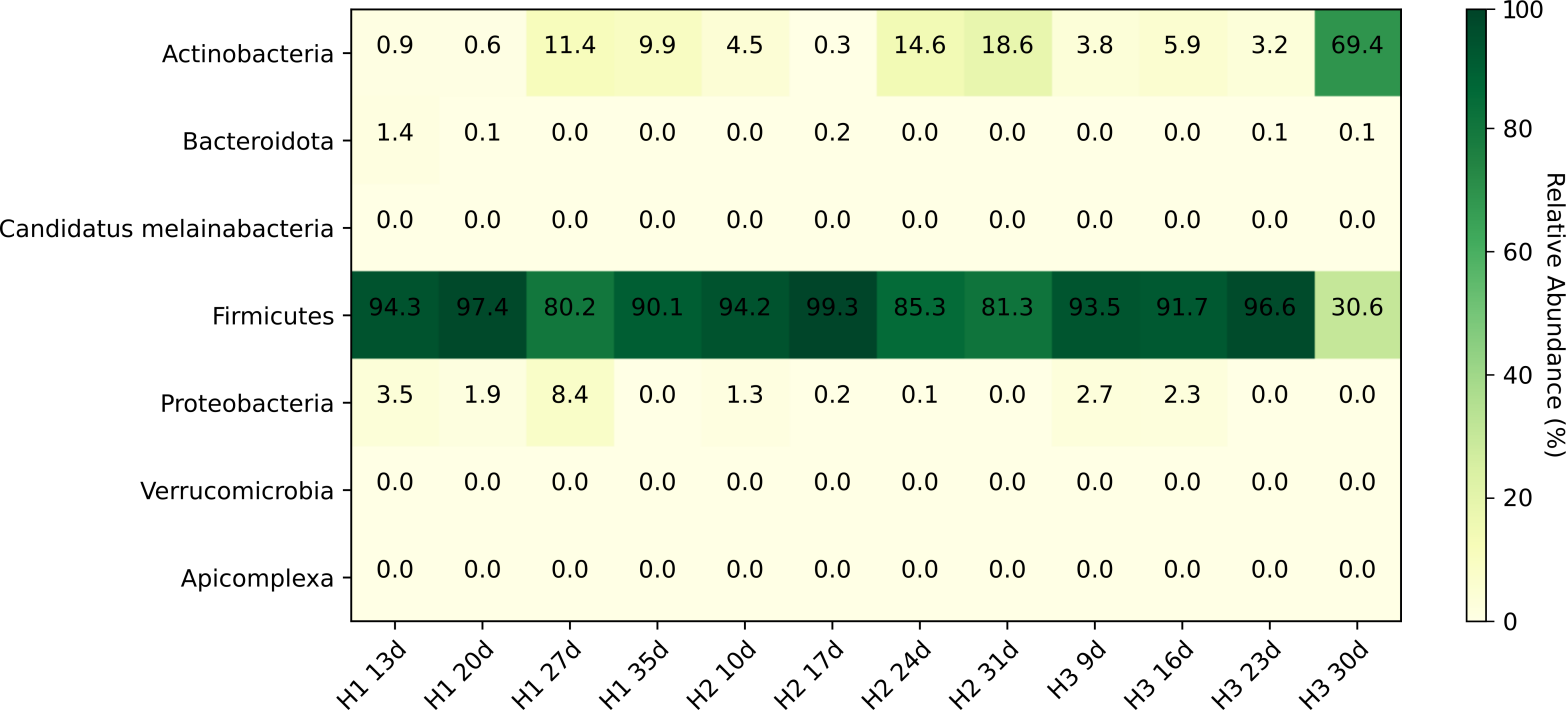
Microbial composition of the samples collected in the chicken farm. Heatmap shows the relative abundance of taxonomic groups (in %) across individual samples based on short-read metagenomic sequencing data.

Based on taxonomic profiling, dominant bacterial taxa within the broiler gut environment were characterized. With the exception of H3_30d, Firmicutes were predominant in most samples, with relative abundance ranging from 80.2% to 99.3% (Fig. 6), indicating a microbial community consistent with the early-life chicken gut ecosystem.

At the species level, the microbiome was primarily composed of lactic acid bacteria and Firmicutes-associated commensals typical for broiler chickens. Dominant taxa included *Lactobacillus johnsonii*, *L. crispatus*, *L. gallinarum*, *Ligilactobacillus aviarius*, *Limosilactobacillus reuteri*, *Weissella jogaejeotgali*, *Enterococcus hirae*, and members of the Lachnospiraceae such as *Blautia* spp. (Table S4). Additional taxa detected at moderate abundance included *Streptococcus alactolyticus*, *Staphylococcus* spp., and *Jeotgalicoccus meleagridis*. Low-abundance representatives of Proteobacteria (*E. coli*) and Bacteroidota (*Bacteroides fragilis*) were detected sporadically across samples. H3_30d was the most distinct from the overall data set, in terms of its resistome, plasmidome, and microbial community structure. Taxonomically, H3_30d was characterized by a predominance of Actinobacteria, comprising 69.4% of the total community, whereas Firmicutes accounted for 30.6% (Fig. 6). Actinobacteria were also detected in other samples; however, the relative abundance ranged from 18.6% to 0.3%. Proteobacteria and Bacteroidota appeared in some samples at low abundances (8.4% to 0.1% and 1.4% to 0.1%, respectively).

## DISCUSSION

This study investigates the plasmidome and metagenome of samples collected from three chicken houses, all of which received enrofloxacin treatment, while house 2 was additionally treated with a trimethoprim-sulfamethoxazole combination. To the best of our knowledge, this is the first study to examine temporal dynamics of horizontal gene transfer using a long-read sequencing approach applied to extracted plasmidome samples.

The assembly of complete plasmids is a challenging task. It was not possible to fully assemble all identified plasmids. On the other hand, this is a first study where a large plasmid was assembled from a whole-community DNA. The assembly incompleteness could be caused by methodological limitations (Supplemental material) or the chimeric nature of the obtained Nanopore reads (28), as observed in the network-based analysis (Fig. 2 and 3) where multiple plasmid groups were co-carried on a single read. However, the complex analysis of long-read sequenced plasmidome revealed plasmid-ARG associations which cannot be analyzed in a short-read metagenomic approach as the sequence depth of the mobilome is not sufficient. The same observations were reported in the previous study where no plasmid-borne ARGs and virulence factors were recovered from metagenome-assembled genomes (29).

Across samples, distinct plasmid-ARG localization patterns were identified. ARGs composing or regulating efflux pumps, such as *acrEF*, *emrAB*, *ermKY,* and other conferring MDR phenotype, were associated with chromosomal fragments (Table S1), which is in concordance with the prevalence presented by Resistance Gene Identifier within CARD database (30). In contrast, fluoroquinolone resistance in our study was strictly linked to *qnr* gene variants on plasmids. Small high-copy *qnr* plasmids as well as a large low-copy plasmid co-carrying *qnr* with a beta-lactamase gene were identified.

The assembled plasmids were highly similar (>99%) to other previously detected plasmids. All of the plasmids similar to fluoroquinolone-associated plasmids from this study were carried by *Enterobacteriaceae*. These were isolated not only from broilers but also from pork, pig feces, wild birds, and human bloodstream infections (31–36), which underscores the potential of these plasmids to circulate among various ecological niches, including livestock, food chain, wildlife, and human population.

The network analysis revealed extensive associations between plasmids and ARGs in the plasmidome. The network was dominated by several highly connected plasmid nodes, primarily belonging to the MOBP group and fluoroquinolone resistance genes. These findings are consistent with previous studies reporting that fluoroquinolone resistance determinants are predominantly plasmid-mediated (37, 38). Within each house, these associations were particularly prominent, consistent with the uniform enrofloxacin treatment administered to all broilers.

Across sampling time points, the distribution of plasmids carrying resistance genes may appear to expand uniformly (Fig. 3). However, examination of the *qnr* abundance profiles shows that each house exhibited increases at different time points which is supported by another study investigating temporal changes of *qnr* levels in poultry after enrofloxacin treatment (38). In contrast, a study (39) conducted on pigs reported the emergence of enrofloxacin-resistant bacteria 35 days after treatment, suggesting that our observation period may have been too short. However, extending the sampling interval was not feasible, as the experiment covered the entire lifespan of broilers up to slaughter.

The strongest association observed in the network involved plasmid CP018223|MOBP and the *qnrB36-like*|pan_9298 gene. Overall, *qnrB36-like|*pan_9298 exhibited a broad plasmid distribution, being detected on more than twenty distinct plasmids across the data set. In contrast, plasmids with the highest overall abundance in the plasmidome (CP022165|MOBP, and NC_002145|MOBP) were not frequently connected to resistance genes.

Variants of *qnr* genes were highly diverse in the plasmidome, where a total of 79 distinct *qnr* variants were detected. On the contrary, only two *qnr* variants were identified in the metagenome, both at very low abundance, demonstrating that plasmid-mediated fluoroquinolone resistance determinants were only weakly represented within the overall microbial community.

Despite the use of sulfamethoxazole/trimethoprim containing treatment in the second chicken house, no substantial increase in *dfr* or *sul* genes prevalence or diversity of plasmid associations was observed. In the plasmidome, a clear association between *dfrD*|pan_8025 and the plasmid NZ_CP009579|MOBV was observed. This confirms *dfr* transfer via plasmids, as did the previous study (40). In the metagenomic data set, the plasmid NZ_CP009579|MOBV carrying the most prevalent *dfr* gene, *dfrD*|pan_8025 showed the highest proportional representations, but its abundance did not reflect the abundance pattern of *dfr* genes. In plasmid samples, we observed increased variability of *dfr* genes in H2, which did not appear in the metagenome. The plasmidome peak detected in H3_30d, largely driven by a single variant dfrD|pan_8025, did not correspond to a similar increase in the metagenome.

High resistance levels and dissemination of *dfr* and *sul* genes were reported after a trimethoprim-sulfamethoxazole treatment in pigs (41) which was stable long after the treatment. Within our study, similar patterns were observed, and the resistance levels and dissemination of *dfr* genes were uniform over time with a slight variability. However, the combined plasmidome and metagenome patterns strongly indicate that the increased resistance levels and the dissemination of *dfr*-carrying plasmids are not treatment-dependent as the increase found in the treated house was not more prominent compared to the untreated houses (Table S3).

The microbial community composition observed in this study largely reflected taxa typical for early-life chicken microbiomes (42), while additional community members were detected with lower abundance. The phylum Firmicutes dominated in most samples (11 out of 12), primarily represented by lactobacilli (*L. johnsonii*, *L. crispatus*, *L. gallinarum*), *Ligilactobacillus* spp., *Limosilactobacilluss* spp., and other lactic acid bacteria. These taxa are known to play central roles in carbohydrate metabolism, colonization resistance, and maintenance of gut homeostasis and commonly dominate early broiler gut succession and immune modulation processes (43).

The Actinobacteria-dominated H3_30d, enriched with taxa such as *Corynebacterium stationis* and *Brachybacterium paraconglomeratum,* likely reflects increased contribution of litter-associated microorganisms (44). In commercial poultry production, chicks are hatched in a sanitized hatchery environment without contact with adult birds, meaning that their initial gut colonization depends solely on microorganisms originating from the surrounding environment (42).

Despite this restricted microbial exposure, we detected a substantial diversity of ARGs, with 473 unique ARGs and resistance to a total of 25 groups of antibiotics identified across samples. Previous metagenomic studies have reported extensive ARG diversity in chicken gut microbiomes and identified commensal taxa including *Lactobacillus*, *Enterococcus*, and *Escherichia* as major ARG hosts (45). However, the diversity observed in our study exceeds the reports of several livestock-associated studies, including a cultivation-based analysis of broiler manure detecting 230 ARGs (7), as well as metagenomic and qPCR investigations identifying 109 ARGs in manure and compost from mixed livestock farms (46), and 201 ARGs in chicken feces (47).

Several methodological limitations should be acknowledged (Supplemental material). Enzymatic removal of chromosomal and linear DNA may reduce the recovery of linearized or fragmented plasmids, potentially leading to an underrepresentation of these elements. Subsequent phi29-based amplification may preferentially enrich small, high copy number plasmids and bias against large, low copy number plasmids (48). However, the use of phi29 DNA polymerase is crucial for obtaining sufficient amounts of pDNA. Nevertheless, long-read plasmidome sequencing enabled the detection of plasmid types that would otherwise remain undetected. These observations underscore the importance of not only filtering out chromosomal sequences prior to plasmid analysis but also extracting plasmids separately as essential steps for their comprehensive characterization. Although the metagenomic approach (7, 49) provides valuable insights into complex microbiomes, including unculturable segments of bacterial communities, plasmids and other MGEs are not adequately sequenced due to the size of the bacterial chromosome (29). Plasmidome analysis allows better resolution of plasmid detection and, thus, better assessment of HGT (12, 16, 17). This approach allowed an identification of multiple *qnr* resistance genes connected to MOBP-like plasmids which would otherwise remain hidden. Furthermore, it was possible to reconstruct complete *qnr*-carrying plasmids from whole-community samples which were highly similar to plasmids originating from diverse sources. Thus, our findings indicate that chicken farms with documented fluoroquinolone exposure may represent important reservoirs of plasmid-mediated antibiotic resistance.

## Data Availability

All raw sequencing reads generated in this study have been deposited in the Sequence Read Archive (SRA) of NCBI under BioProject accession number PRJNA1227156. Individual annotated plasmids are available under accession numbers PX387656–PX387661, PX387668–PX387670, and PX399214–PX399216. All scripts used within the manuscript are publicly available within a GitHub repository (https://github.com/janpal-cz/Plasmidome).

## SUPPLEMENTAL MATERIAL

Figure S1 spectrum.04074-25-s0001.docx Co-occurrence network of antibiotic resistance genes and plasmids.

Figure S2 spectrum.04074-25-s0002.docx Top 10 most abundant mobile genetic elements (MGEs) detected across samples.

Figure S3 spectrum.04074-25-s0003.docx Total plasmid abundance across samples from different houses and time points.

Figure S4 spectrum.04074-25-s0004.docx Overview of quality control and sequencing statistics for plasmidome samples across the chicken farm.

Supplemental material spectrum.04074-25-s0005.docx Supplemental methods, results, and potential technical limitations.

Table S1 spectrum.04074-25-s0006.xlsx Assembly metadata obtained from analysis of plasmid and antibiotic resistance genes content.

Table S2 spectrum.04074-25-s0007.xlsx Linking plasmids and resistance genes.

Table S3 spectrum.04074-25-s0008.xlsx Total abundance of ARGs, MGE, and plasmids.

Table S4 spectrum.04074-25-s0009.xlsx Species-level taxonomy.

Table S5 spectrum.04074-25-s0010.xlsx Overview of sequencing statistics.

ASM does not own the copyrights to Supplemental Material that may be linked to, or accessed through, an article. The authors have granted ASM a non-exclusive, world-wide license to publish the Supplemental Material files. Please contact the corresponding author directly for reuse.
