# Supplementary material for: Dynamic changes in the plasmidome and resistome in the gastrointestinal tract of chickens
Source: Microbiol Spectr. 2026 Mar 26;14(5):e04074-25. doi: 10.1128/spectrum.04074-25 (PMC13142040; doi:10.1128/spectrum.04074-25)
Supplement: Figure S1 — Co-occurrence network of antibiotic resistance genes and plasmids. [file spectrum.04074-25-s0001.docx]

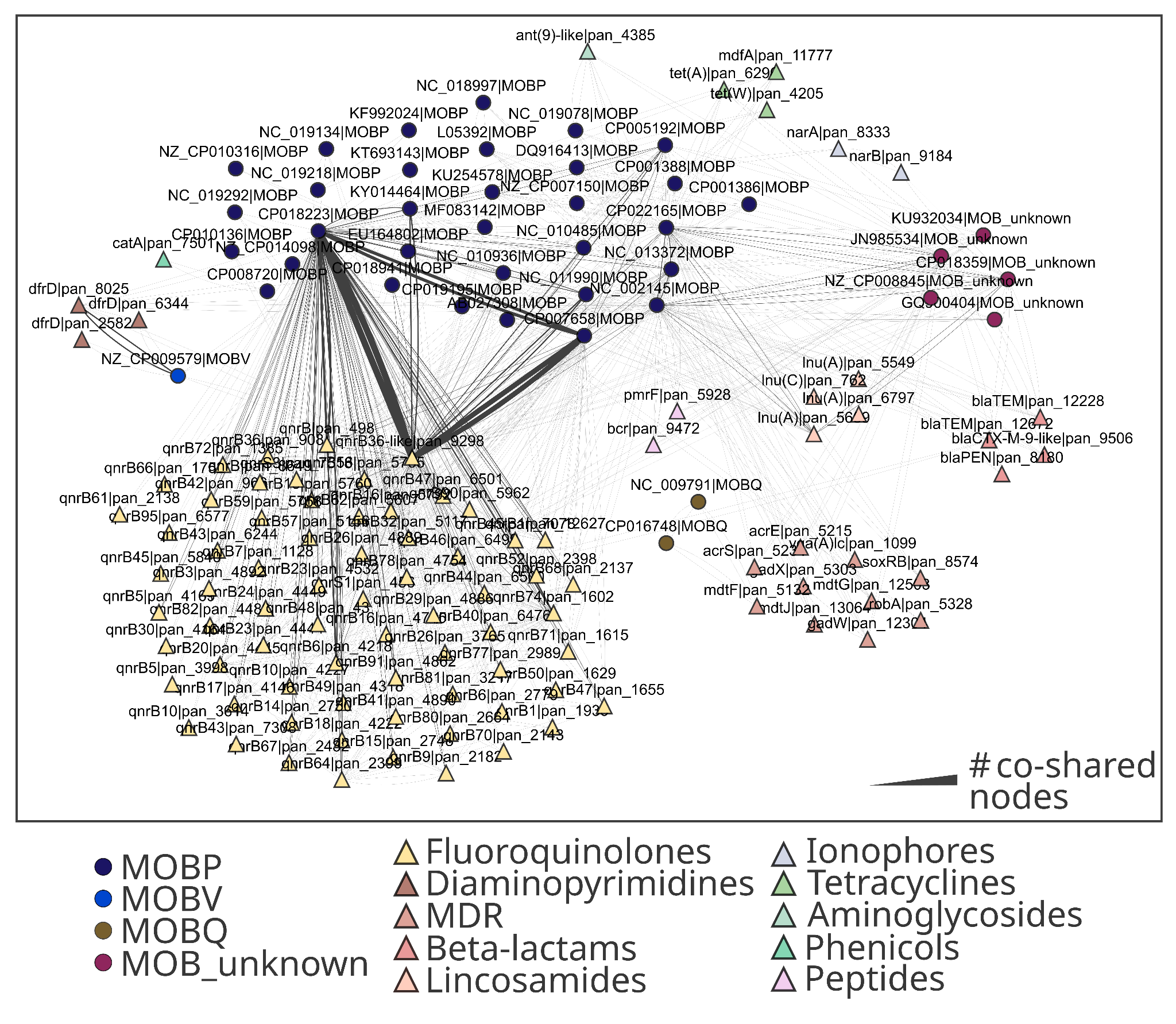
**Fig.S1. Co-occurrence network of antibiotic resistance genes and plasmids**

The undirected network consists of 141 nodes and 858 edges, where nodes represent plasmids (circles) or antibiotic resistance genes (triangles), and edges represent their co-occurrence on the same sequence. The network is divided into several disconnected components (7 connected components), forming distinct yet closely connected clusters (clustering coefficient 0.69) with nodes within clusters exhibiting a compact structure (characteristic path length 2.4). Even though the overall connectivity is relatively sparse (network density 0.05), most nodes are linked to multiple nodes (average number of neighbours 6.5).

Layout does not represent similarity or spatial distance as node positions were manually adjusted for an improved visual clarity.
