# Supplementary material for: Dynamic changes in the plasmidome and resistome in the gastrointestinal tract of chickens
Source: Microbiol Spectr. 2026 Mar 26;14(5):e04074-25. doi: 10.1128/spectrum.04074-25 (PMC13142040; doi:10.1128/spectrum.04074-25)
Supplement: Figure S2 — Top 10 most abundant mobile genetic elements (MGEs) detected across samples. [file spectrum.04074-25-s0002.docx]

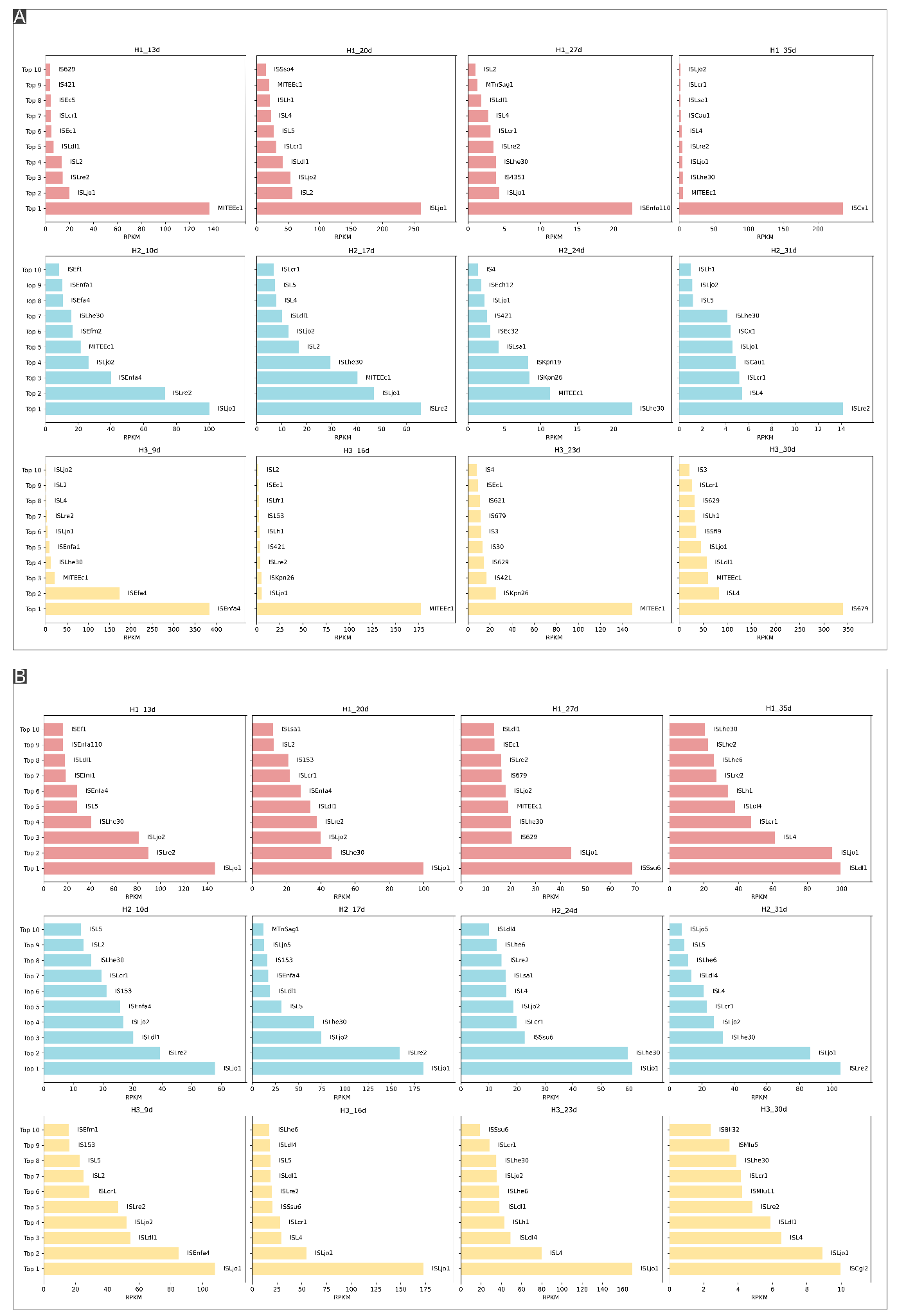


**Fig. S2. Top 10 most abundant mobile genetic elements (MGEs) detected across samples.**

Panel A shows the most abundant MGEs based on long-read sequencing (plasmidome), while panel B shows those detected using short-read sequencing (metagenome). MGEs are ranked by RPKM (Reads per kilobase million) values for each sample. Each barplot represents one sample, organized by house and sampling time point. The two panels are in different scales. The top 20 MGEs were categorized separately, remaining elements were grouped as ‘others’.
