# Supplementary material for: Dynamic changes in the plasmidome and resistome in the gastrointestinal tract of chickens
Source: Microbiol Spectr. 2026 Mar 26;14(5):e04074-25. doi: 10.1128/spectrum.04074-25 (PMC13142040; doi:10.1128/spectrum.04074-25)
Supplement: Figure S3 — Total plasmid abundance across samples from different houses and time points. [file spectrum.04074-25-s0003.docx]

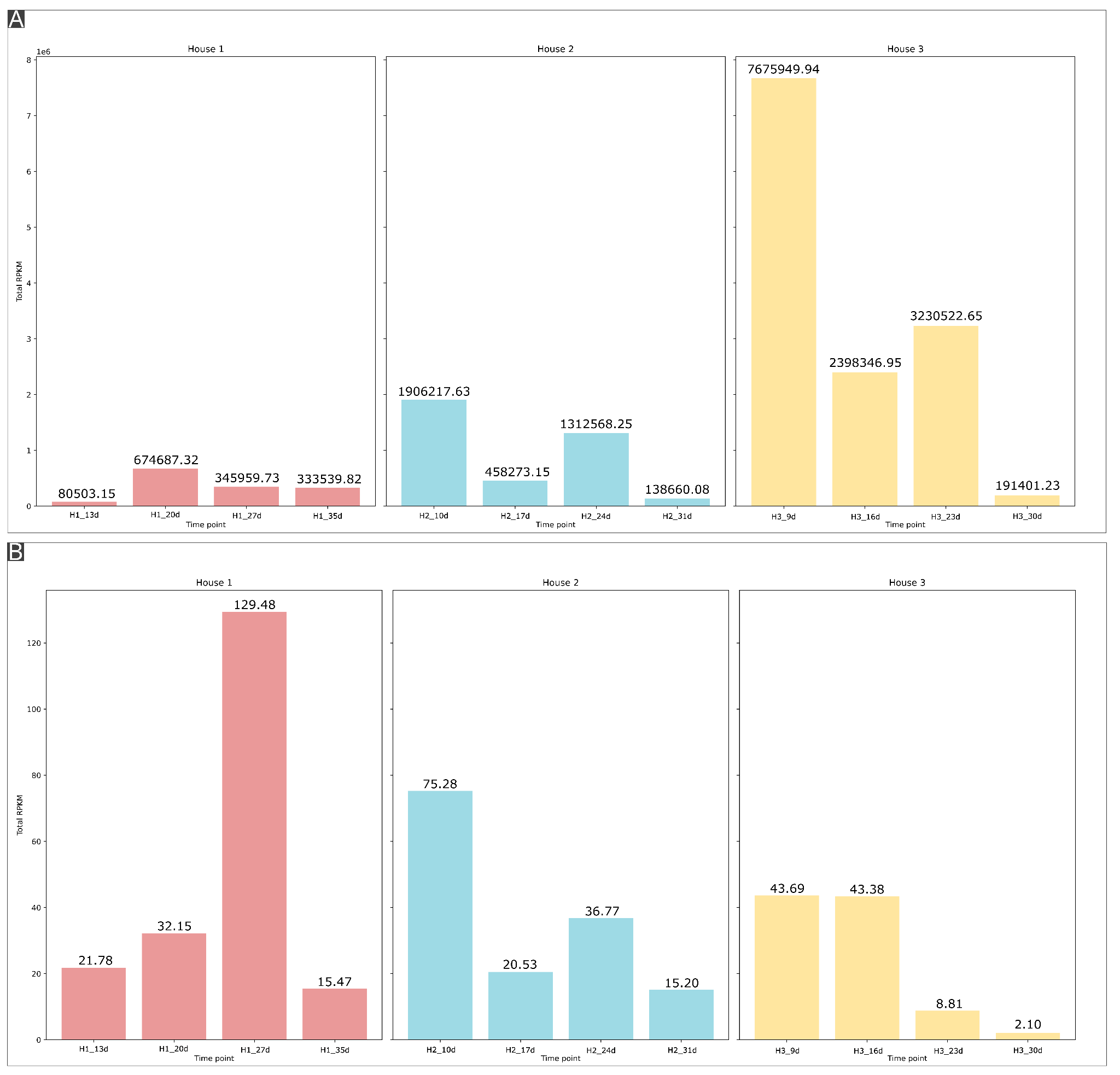


**Fig. S3. Total plasmid abundance across samples from different houses and time points.**

Total plasmid abundance is shown for the plasmidome based on long-read sequencing in panel A, and for the metagenome based on short-read sequencing in panel B. Samples are organized by house and sampling time point, and abundance is expressed in RPKM (Reads per kilobase million). The two panels are in different scales.
