# Supplementary material for: Dynamic changes in the plasmidome and resistome in the gastrointestinal tract of chickens
Source: Microbiol Spectr. 2026 Mar 26;14(5):e04074-25. doi: 10.1128/spectrum.04074-25 (PMC13142040; doi:10.1128/spectrum.04074-25)
Supplement: Figure S4 — Overview of quality control and sequencing statistics for plasmidome samples across the chicken farm. [file spectrum.04074-25-s0004.docx]

**
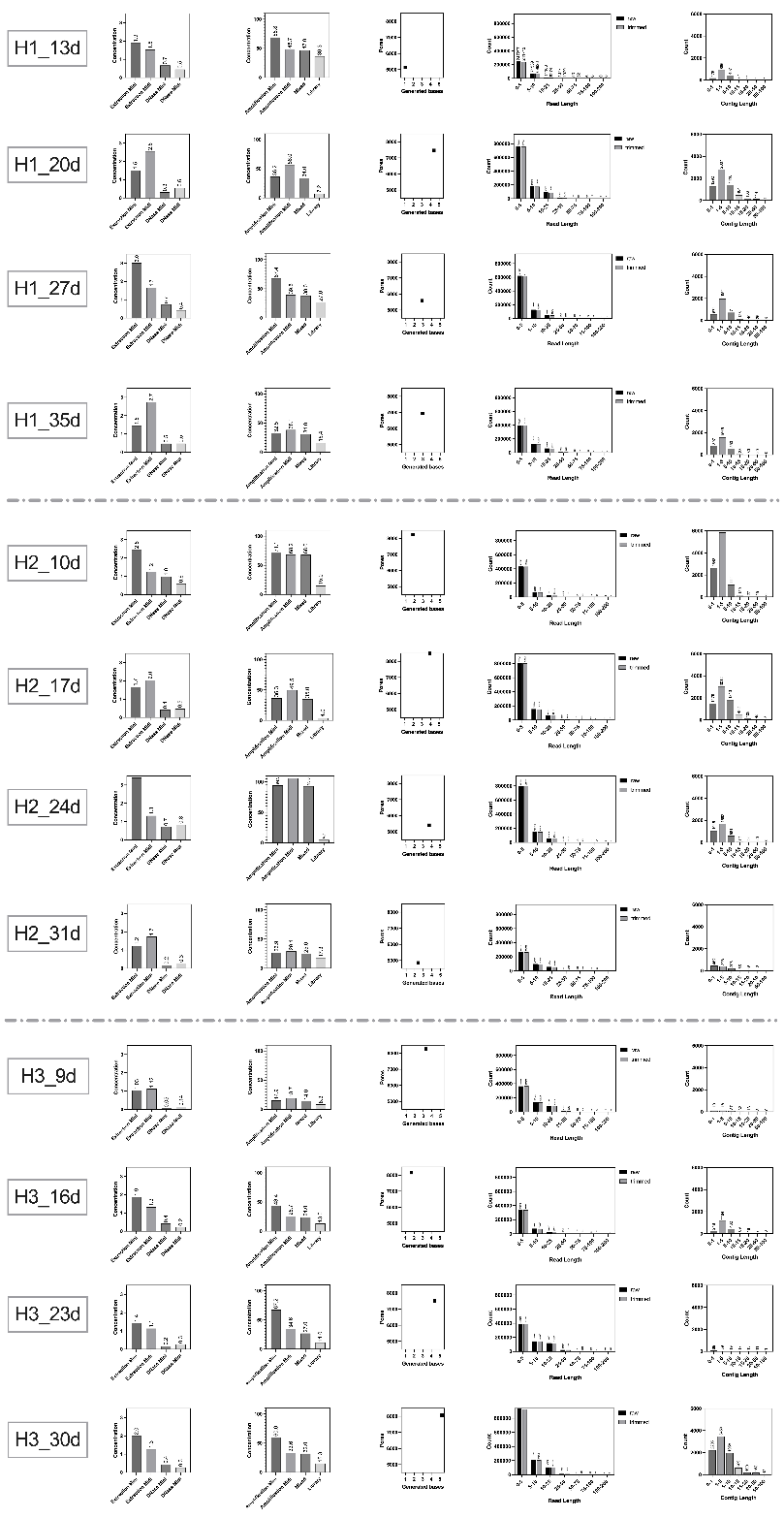
**

**Fig. S4: Overview of quality control and sequencing statistics for plasmidome samples across the chicken farm.** Each row represents one sample collected from a specific house and time point. The columns show (from left to right): DNA concentration after extraction and DNAse treatment (ng/μL), DNA concentration after amplification and library preparation (ng/μL), number of generated bases (Gb), read length (kb) distribution (raw vs. trimmed), and contig length (kb) distribution after assembly.
