# Supplementary material for: Dynamic changes in the plasmidome and resistome in the gastrointestinal tract of chickens
Source: Microbiol Spectr. 2026 Mar 26;14(5):e04074-25. doi: 10.1128/spectrum.04074-25 (PMC13142040; doi:10.1128/spectrum.04074-25)
Supplement: Supplemental material — Supplemental methods, results, and potential technical limitations. [file spectrum.04074-25-s0005.docx]

**Supplementary material**

**Methods**

**DNA extraction and post-extraction treatment for long-read sequencing**

*DNA extraction*

Before extraction, 100 mg of each sample was subjected to a 30 min incubation at 37°C and 185 rpm in 1.5 mL sodium dodecyl sulfate (SDS, Sigma-Aldrich, USA). For a better cell wall dissolution, 200 µL of phosphate buffered saline (Thermo Fisher Scientific, USA) containing lysozyme from chicken egg (Sigma-Aldrich, USA) in concentration 100 mg/mL was added to each sample in 2 mL of buffer P1 (Qiagen, GE). All buffer volumes were adjusted to the sample weight 0.1 grams. Protein precipitation with P3 buffer mixture was incubated on ice for 20 min, elution buffer QF was preheated at 65°C prior to application, and the pDNA pellet washing step was performed using ice-cold 70% ethanol after isopropanol precipitation. The pDNA pellet was dissolved in 50 µL and 200 µL of TE buffer (Thermo Fisher Scientific, USA) for Plasmid Mini Kit and Plasmid Midi Kit, respectively.

*Post-extraction treatment*

A total of 42 µL of pDNA was treated with Plasmid-Safe ATP-Dependent DNase (10 U) (Biosearch Technologies, UK) to remove environmental contaminants and bacterial chromosomal DNA. The pDNA from the Plasmid Mini Kit and the Plasmid Midi Kit were treated separately. The mixture of pDNA and other components from the kit (2 µL of 25 mM ATP, 5 µL of 10x Reaction Buffer, 1 µL of Plasmid-Safe ATP-Dependent DNase) was incubated at 37°C for 16 hours, followed by enzyme inactivation at 70°C for 30 minutes. Treated DNA was amplified with NxGen phi29 DNA Polymerase (10 U) (Biosearch Technologies, UK) in triplicates to obtain sufficient quantity of pDNA for sequencing. The mixture (6 µL of treated denatured pDNA, 3 µL of 2.5 mM dNTP mix, 3 µL of 100 μM Random hexamer primers (Thermo Fisher Scientific, USA)) was added to 48 µL of phi29 mix (36 µL of water, 6 µL of 10x phi29 buffer and 6 µL of NxGen phi29 DNA polymerase) and incubated at 30°C for 16 hours, followed by enzyme inactivation at 65°C for 10 min. DNA yield was evaluated after extraction, DNase treatment and amplification using the dsDNA BR and HS assay kit on a Qubit fluorometer (Invitrogen, USA). All negative controls exhibited undetectable levels of DNA.

**Plasmidome and Metagenome Sequencing**

The following modifications were employed for DNA library preparation using SQK-LSK114 ligation kit (Oxford Nanopore Technologies, UK). After the end repair and dA-tailing steps, incubation was extended to 10 min at 20°C and 10 min at 65°C. Each sample mixer incubation was prolonged to 10 min and incubation in the ligation step was extended to 40 min. Long fragment buffer (LFB) was used to remove short DNA fragments (<3 kb).

**Long-read data analysis**

Raw data obtained from long-read sequencing were adapter trimmed using Porechop v0.2.4 (1). Subsequently, reads of low quality (Q ≤ 9) and length (read length < 200 bp) were removed with BBduk from BBmap package v39.01 (sourceforge.net/projects/bbmap/). The quality control of reads was performed prior and after quality and length trimming using FastQC v0.12.1(2). GraphPad Prism v10.0.0 (GraphPad Software, Boston, USA) was used to analyze the associations between concentration, number of flow cell pores, number of generated data, read lengths and lengths of assembled contigs. The total length of reads was normalized. The statistical analysis was performed using Pearson correlation, with a significance threshold set at *p* ≤ 0.05.

PanRes v1.0.1 database used for ARGs identification incorporates 8 different databases (ResFinder, ResFinder FG, CARD, MegaRes, AMRFinderPlus, ARGANNOT, The ‘CsabaPal’ collection and BacMet) (3). The analysis was focused on the ARGs (excluding metals and biocides) and the hits from The ‘CsabaPal’ collection were discarded, due to insufficient information from this collection. The threshold for coverage and identity was set to 80% and 90%, respectively. The final gene name was created using a gene annotation and pan number in the form ‘gene|pan number’ to obtain an informative result.

A custom Python script was used to identify reads containing both gene types within the datasets. Unique genes were extracted, and a binary presence-absence matrix was constructed for each read. A gene transfer matrix was computed to assess the extent of gene co-sharing across samples, following the previously described approach (4).

Plasmids carrying ARGs were assembled using Flye v2.9.5 with the ‘meta’ option (5). Obtained assemblies were error-corrected by Racon v1.5.0 (6) and the contigs with length < 200 bp were filtered out. The assemblies were analyzed using the reconstruction mode of MOB-suite (7) to obtain information about sequence type, plasmid replicon, relaxase, mate-pair formation type and transfer type. The contigs typed as a plasmid or containing one of the plasmid markers (detection of plasmid replicon, relaxase, mate-pair formation or plasmid *oriT*) were considered plasmids.

**Short-read data analysis**

Short shotgun metagenomic sequencing reads were trimmed using fastp v0.23.4 (8) with default settings. Human-origin DNA was then removed using BBMap v39.01 with the GCF_000001405.40 genome from the NCBI RefSeq database. Such trimmed and contamination-free short read sequences were used for the downstream analyses.

**Results**

**Potential technical limitations in plasmidome analysis**

Despite the strengths of this study, several methodological limitations should be acknowledged. Although plasmid extraction procedures, DNase treatment, and phi29-mediated amplification of circular DNA were employed, residual chromosomal DNA fragments were still detected in the samples. The treatment of extracted pDNA with Plasmid-Safe ATP-dependent DNase, intended to remove chromosomal and linear environmental DNA, may have inadvertently degraded linearized or fragmented plasmids which could have contributed to a selective loss of large, low-copy-number plasmids. The use of phi29 DNA polymerase for plasmid DNA amplification, which exhibits preferential amplification of small DNA templates, potentially leads to the overrepresentation of small high copy number plasmids. Moreover, the phi29 amplification approach generates tandem repeats of the target plasmid, as confirmed by restriction digestion (9). However, the use of phi29 DNA polymerase is crucial for obtaining sufficient amounts of pDNA and all samples were processed identically, therefore, any amplification bias introduced by phi29 DNA polymerase is expected to affect all samples in a comparable manner.

The processing of sequencing data largely depending on databases of known genes can also represent a limitation of the current research, as well as the use of fixed thresholds to define gene presence. As a result, novel or highly divergent resistance determinants may remain undetected. However, these limitations are common to many studies relying on sequence-based detection of genes and were recognized in the previous research (10, 11). And even though the study is dependent on the known genes, our study benefits from comprehensive, up-to-date resources, such as PanRes database which integrates major ARGs databases, or PLSDB, the largest curated repository of complete plasmid sequences.

**Association of pore count and data quality**

To better understand potential technical limitations associated with plasmidome sequencing, the influence of sequencing parameters on dataset characteristics was evaluated.

Pearson correlation analysis revealed a weak positive relationship between the number of pores on the flow cell and the number of generated bases (*r* = 0.337, *p* = 0.286) (Fig. S4, Table S1). While this suggests a tendency for a higher pore count to result in increased base output, the correlation was not statistically significant. In contrast, a moderate negative correlation was observed between the pDNA concentration after the second wash and the number of generated bases (r = -0.590, p = 0.043). Although the correlation is not strong, it indicates that higher DNA concentrations on the flow cell may be associated with reduced sequencing efficiency.

A highly significant, strong linear relationship exists between the amount of generated data and total read length, both with raw untrimmed (r = 0.9979, *p* = 0.00001) and trimmed (r = 0.9983, *p* = 0.00001) sequencing data. This confirms that a higher number of generated bases corresponds to longer reads, suggesting that increasing sequencing output leads to an overall increase in read length. In contrast, the number of generated bases did not significantly affect either the number of contigs (*r* = 0.28, *p* = 0.38) or their total length (*r* = 0.4573, *p* = 0.13). Similarly, total read length had no significant impact on contig length (*r* = 0.48, *p* = 0.11) or the number of contigs (*r* = 0.291, *p* = 0.36). All data is available in the supplementary material (Table S5).

These results highlight that while some technical parameters, such as total read length predictably influence sequencing output, others, including flow cell pore count or input DNA concentration, may have unexpected or minimal effects.

**References**

1. Wick RR, Judd LM, Gorrie CL, Holt KE. 2017. Completing bacterial genome assemblies with multiplex MinION sequencing. Microb Genom 3:e000132.

2. Babraham Bioinformatics - FastQC A Quality Control tool for High Throughput Sequence Data. https://www.bioinformatics.babraham.ac.uk/projects/fastqc/. Retrieved 6 May 2025.

3. Martiny H-M, Pyrounakis N, Lukjančenko O, Petersen TN, Aarestrup FM, Clausen PTLC, Munk P. PanRes - Collection of antimicrobial resistance genes https://doi.org/10.5281/ZENODO.10091602.

4. Schwarzerova J, Rajasekaran L, Jureckova K, Nejezchlebova J, Varga M, Provaznik V, Weckwerth W, Cejkova D. 2024. Visualizing Horizontal Gene Transfer Detection in Phylogenetically Divergent Bacteria. Lecture Notes in Computer Science (including subseries Lecture Notes in Artificial Intelligence and Lecture Notes in Bioinformatics) 14849 LNBI:267–277.

5. Kolmogorov M, Bickhart DM, Behsaz B, Gurevich A, Rayko M, Shin SB, Kuhn K, Yuan J, Polevikov E, Smith TPL, Pevzner PA. 2020. metaFlye: scalable long-read metagenome assembly using repeat graphs. Nat Methods 17:1103–1110.

6. Vaser R, Sović I, Nagarajan N, Šikić M. 2017. Fast and accurate de novo genome assembly from long uncorrected reads. Genome Res 27:737–746.

7. Robertson J, Nash JHE. 2018. MOB-suite: software tools for clustering, reconstruction and typing of plasmids from draft assemblies. Microb Genom 4:e000206.

8. Chen S. 2023. Ultrafast one-pass FASTQ data preprocessing, quality control, and deduplication using fastp. iMeta 2:e107.

9. Dean FB, Nelson JR, Giesler TL, Lasken RS. 2001. Rapid Amplification of Plasmid and Phage DNA Using Phi29 DNA Polymerase and Multiply-Primed Rolling Circle Amplification. Genome Res 11:1095–1099.

10. Papp M, Solymosi N. 2022. Review and Comparison of Antimicrobial Resistance Gene Databases. Antibiotics 11:339.

11. Gschwind R, Ugarcina Perovic S, Weiss M, Petitjean M, Lao J, Coelho LP, Ruppé E. 2023. ResFinderFG v2.0: a database of antibiotic resistance genes obtained by functional metagenomics. Nucleic Acids Res 51:W493.
